# The Many Faces of Glioblastoma-Associated Macrophages and Their Gene Signatures

**DOI:** 10.3390/cancers18040624

**Published:** 2026-02-14

**Authors:** Mikael S. Lindström

**Affiliations:** Science for Life Laboratory, Division of Genome Biology, Department of Medical Biochemistry and Biophysics, Karolinska Institutet, SE-171 77 Stockholm, Sweden; mikael.lindstrom@ki.se

## 1. A Five-Gene Macrophage Signature for GBM Patient Outcomes

The prognosis for high-grade gliomas, particularly glioblastoma (GBM), remains dismal. In a recent study, Chen et al. defined a concise five-gene “Macrophage-Associated Prognostic Signature” (MAPS), comprising *THEMIS2*, *SIGLEC9*, *CSTA*, *LILRB3*, and *MS4A6A* [1]. The MAPS stratifies patients into high- and low-risk groups, linking macrophage-specific expression to poor prognostic features.

Among several obstacles impeding the efficacy of novel therapies in GBM are the blood–brain barrier and the immunosuppressive T-cell cold tumor microenvironment (TME) [2,3]. Perhaps counterintuitive to the idea that GBM is a cold tumor is the fact that it is often rich in diverse innate immune cells [4,5]. This hypoxic, immunosuppressive TME in GBM is dominated by TAMs, which constitute a heterogeneous population originating from resident microglia and monocytes (bone marrow-derived macrophages) [5,6,7]. TAMs are plastic and capable of expressing a variety of surface markers and receptors [8]. However, TAMs can be reprogrammed by GBM cells, shaping the microenvironment toward an immunosuppressed state with poor prognostic features [9,10,11].

Although the study by Chen et al. is an in silico, data analysis-driven study, there are interesting aspects of the study design, including a high level of bioinformatic rigor. The research team derived MAPS by integrative analysis of bulk RNA-seq from multiple cohorts and single-cell RNA-seq (scRNA-seq) data from GBM. The use of independent GBM datasets increases confidence in the robustness of the signature. While MAPS was trained and tested on existing datasets, it still needs to be further validated and investigated in prospective clinical trials or in new patient samples. A primary challenge for any new biomarker in GBM is to demonstrate added value over established clinical markers. The performance of MAPS was modest, indicating that it is not, on its own, a perfect prognostic tool and it needs to be combined with other clinical factors to achieve higher accuracy, for example, standard markers such as *MGMT* promoter methylation or *IDH* status [12].

However, the clinical value of MAPS lies in its potential to guide personalized treatment, for example, combinatorial immunotherapy, and tailored drug-sensitivity studies. Furthermore, the signature provides several important insights into aspects of GBM immunobiology, supported by recent literature. The study complements previous single-cell RNA-seq analyses [10,13] and is consistent with the notion of multiple TAM activation states and phenotypes.

MAPS-high tumors were characterized by higher immune and stromal scores and upregulation of inhibitory immune checkpoints, including *PDCD1* and *CSF1R* [1]. Differential gene expression analysis between high- and low-risk groups revealed enrichment of cytokine-signaling pathways and leukocyte activation in MAPS-high tumors, hallmarks of an active but likely dysfunctional immune environment. In a GBM single-cell dataset, all five MAPS genes were expressed in macrophage-lineage cells, and subclustering of these macrophages showed that the MAPS genes are particularly highly expressed in proliferative TAMs and TAM subtypes involved in tumor support [1]. Identification of a proliferative TAM cluster (marked by *MKI67*, *STMN1*, and *TYMS*) is notable and consistent with previous observations of cycling myeloid states in glioma and other tumors [14,15]. Also of interest are transitional states, with implications for the understanding of macrophage plasticity. Spatial analysis using the Ivy Glioblastoma Atlas Project demonstrated that four of the five genes (*CSTA*, *SIGLEC9*, *LILRB3*, and *MS4A6A*) are expressed in angiogenic and hypoxic niches [1]. This suggests that MAPS is reflecting a macrophage program linked to angiogenesis and immune evasion. Interestingly, a recent study revealed the existence of a hypoxic macrophage population producing adrenomedullin, and that targeting this axis normalizes vasculature and improves drug delivery [16], in support of the MAPS angiogenic/hypoxic signal.

As MAPS-high tumors are characterized by an immunosuppressed milieu, it can be anticipated that such tumors might not respond well to checkpoint blockade alone. Instead, the authors proposed combining TAM-directed treatments (e.g., CSF1R inhibitors) with checkpoint inhibitors to reawaken antitumor immunity [1]. Unfortunately, CSF1R inhibitors have largely failed in GBM trials, with effects of lineage replacement, compensatory recruitment, and phenotypic reprogramming of TAMs seen in the TME [17,18], indicating that different combinatorial approaches or functional endpoints are needed. Low-MAPS patients, on the other hand, might be directed toward different targeted therapies.

Indeed, beyond risk prognosis, the team modeled therapeutic implications. Using in silico transcriptomic drug-sensitivity models, they found that MAPS-high tumors are predicted to be more sensitive to inhibitors of the IGF-1R/MAPK pathway (e.g., BMS-536924, an IGF-1R inhibitor; SCH772984, an ERK1/2 inhibitor; selumetinib, a MEK1/2 inhibitor) [1]. These drugs also target angiogenic signaling, consistent with the MAPS-high angiogenic signature. In contrast, MAPS-low tumors were predicted to respond better to other agents (e.g., the aurora kinase inhibitor tozasertib, HDAC8 inhibitor PCI-34051, vincristine, and the survivin inhibitor sepantronium bromide) [1]. While in silico predictions of drug sensitivity are rather hypothetical and unreliable for clinical translation [19], and most of these drugs are unlikely to be clinically implemented in GBM due to the fact that only a tiny fraction of small-molecule anticancer agents can effectively pass the blood–brain barrier to achieve therapeutically relevant concentration [20], the findings raise some hypotheses about tailoring therapy to MAPS status.

MAPS genes might simply be markers of tumor burden or hypoxia rather than functionally important players. However, the MAPS gene signature is surprisingly informative. Central to the signature are *SIGLEC9* and *LILRB3*, inhibitory receptors that translate tumor sialylation and lipid-rich necrosis into suppressive cellular signaling [21,22,23]. *SIGLEC9* functions as a myeloid-side checkpoint that restricts T-cell priming, and its expression in TAMs appears to be a primary driver of resistance in anti-PD-1 non-responders [24]. *LILRB3* may link the unique lipid metabolism of GBM to immune evasion by binding apolipoprotein E, particularly in the aggressive mesenchymal cellular state [22]. *THEMIS2* complements this by acting as an intracellular signaling scaffold that modulates Toll-like receptor and cytokine outputs, effectively stabilizing the suppressive TAM phenotype [25].

As the authors note, the links between *THEMIS2*, *SIGLEC9*, and macrophage behavior remain to be fully elucidated. For example, knocking down *THEMIS2* could reveal whether it is in fact involved in angiogenesis or immunosuppression. Similarly, co-culture of GBM cells with MAPS-high versus MAPS-low macrophages could test causal effects on tumor growth or T-cell function. Spatial omics and imaging could also be further developed; for instance, high-resolution spatial analyses could map MAPS gene expression in situ, visualizing interactions between macrophages, blood vessels, neurons, and GBM cells.

## 2. Understanding Differences in TAM Gene Signature Panels

Chen et al.’s findings fit into a broader picture in which TAMs dominate the GBM immunobiology. Indeed, TAMs can comprise 20–40% of the cells within a tumor [4,26]. They secrete cytokines, chemokines, and growth factors that promote glioma cell stemness, proliferation, migration, angiogenesis, and further immune evasion [9,26]. Previous studies have also derived TAM-related gene signature of survival outcomes, although these often differ due to initial assumptions and selections [27,28,29,30,31]. The new MAPS builds on these results by implicating macrophage biology even more but is notable for its brevity (only five genes) and for anchoring each component to macrophage subtypes.

Why do macrophage gene signatures differ and sometimes show inconsistent prognostic value? What biological or methodological factors drive these discrepancies? On one hand, discordance between signatures might be informative. TAMs are heterogeneous, they occupy different spatial niches such as the hypoxic core, perivascular niche, and invasive margin [32]. As mentioned earlier, hypoxic macrophage states have been characterized [16,33]. Recently, a group of lipid-loaded macrophages was described in GBM, and that may constitute a relevant therapeutic target [34]. The gene signatures are likely capturing different aspects of this macrophage biology reflecting distinct cell ontogenies, spatial niches, and functional states. Tumor subtype also plays a role: mesenchymal GBMs have different TAM compositions than other subtypes [35]. Moreover, tumor evolution and prior therapy reshape the TAM landscape. An important limitation of current TAM signatures is that they implicitly treat macrophage heterogeneity as a collection of relatively stable cell states. GBM-associated macrophages may continuously traverse transcriptional and functional trajectories constrained by spatial and metabolic cues [14,36,37,38].

Another important aspect is the reciprocal relationship between resident microglia and the infiltrating macrophages. Blocking the recruitment of macrophages causes a reciprocal increase in the density of resident microglia and vice versa [39], and this plasticity may be involved in therapeutic failure. Balance or competition between different myeloid populations in GBM needs to be considered in future biomarker and therapy development. Signatures that primarily mark monocyte-derived states may then only tell half the story.

On the other hand, some of the differences reported in the literature likely stem from methodological factors, rather than biology, including variations in cell-type deconvolution algorithms, batch effects in multi-cohort integration, initial selections, and choice of statistical cutoffs and corrections [40,41]. Bulk-derived signatures may correlate with survival because of tumor purity, and as mentioned mesenchymal subtype fraction, as well as the extent of stromal infiltration [42]. Without spatial context, bulk signatures cannot fully capture which macrophages are being measured. Thus, the field has generated conflicting signatures because aspects of macrophage biology are being measured in different contexts. With increased technical robustness in single cell omics and spatial protein and RNA technologies, we are likely to see improved gene signatures and biomarkers.

## 3. Conclusions

In summary, the study presents a concise macrophage-based signature that highlights important aspects of GBM immune biology and patient prognosis. This MAPS panel emphasizes that TAM-driven pathways of angiogenesis and immunosuppression have a significant impact on GBM outcomes. MAPS and similar gene signatures provide a useful way of thinking for personalized therapy, as they flag tumors that are macrophage-high and may require aggressive TAM-directed strategies.

As a personal reflection, we now understand the biology of GBM better than ever before. We are making progress and developing increasingly sophisticated tools to dissect GBM biology, but these tools are being generated faster than they can be validated or implemented in clinical practice. The insight from studies like this may therefore reside more in the biology itself and in the recognition that GBM harbors multiple functionally divergent but coexisting macrophage populations. These macrophages, in turn, interact in different ways with GBM and normal cells. Future biomarkers will have to capture this complexity. Until then, MAPS and similar signatures remain valuable research tools, awaiting clinical validation to distinguish actionable findings from elegant but ultimately limited bioinformatic predictions.

## Data Availability

No new data were created.
